# Characteristics of continental shale reservoirs in the Xuanhua Basin of Yanshan area and their influence on gas-bearing properties

**DOI:** 10.1038/s41598-025-30550-z

**Published:** 2025-12-01

**Authors:** Wei Jiang, Jiasheng Geng, Wenheng Hu, Yang Hu, Gang Liu

**Affiliations:** 1 School of Resources and Civil Engineering, Suzhou University, Suzhou, 234000 China; 2 Key Laboratory of Coalbed Methane Development and Mining Area Environmental Remediation of Suzhou, School of Resources and Civil Engineering, Suzhou University, Suzhou, 234000 China; 3 Anhui Hengyuan Coal and Electricity Group Company Limited, Renlou Coal Mine, Huaibei, 235123 China

**Keywords:** Xuanhua basin, Xiahuayuan formation, Continental shale, Gas-bearing properties, Main controlling factors, Energy science and technology, Solid Earth sciences

## Abstract

The continental shale of the Jurassic Xiahuayuan Formation in the Xuanhua Basin of the Yanshan area displays widespread occurrence and notable thickness, indicating good potential for industrial-scale shale gas production. However, there are still many uncertainties regarding the characteristics of shale reservoirs, their gas-bearing properties, and the main controlling factors. To characterize the Xiahuayuan Formation shale, this study employed total organic carbon (TOC) analysis, X-ray diffraction (XRD), low-temperature N_2_ adsorption (LTN_2_A), and in-situ gas content measurement to systematically investigate organic matter (OM) composition, reservoir properties, and gas occurrence characteristics. Moreover, the principal influencing factors of the gas-bearing characteristics were discussed. The results indicate that the Xiahuayuan Formation shale exhibits relatively high TOC content and is in the mature to high-mature stage. The OM type is mainly Type Ⅲ kerogen, providing a favorable foundation for the formation and storage of shale gas. The in-situ desorption gas content is predominantly composed of free gas. Both the adsorbed and free gas contents gradually increase as burial depth increases. Shale gas content is influenced by multiple geological factors. Among them, the TOC is conducive to hydrocarbon generation in shale. It promotes the development of OM pores, increases pore volume (PV) and specific surface area (SSA), and thus shows a positive correlation with various gas contents. As R_o_ increases, both the hydrocarbon adsorption capacity and the ability to store free gas are enhanced. In addition, quartz has a remarkable resistance to compaction and can effectively protect the pore structure. When its content is relatively high, it tends to have a slight positive relationship with various gas contents. Conversely, carbonate minerals, by filling the original pores and microfractures, show a negative association with various gas contents. In contrast, clay minerals contribute to the formation of numerous secondary pores and show a positive correlation with various gas contents. The pore structure of shale primarily consists of mesopores and micropores. An increase in micropore volume leads to a reduction in the average pore size (APS), promoting an increase in SSA and PV, and further enhancing the adsorption capacity and storage space of shale. This research are intended to offer a robust geological foundation for future exploration and development efforts related to continental shale gas resources.

## Introduction

Shale gas, an unconventional natural hydrocarbon resource, is generated and stored within its source formation and accumulates in situ. It has great potential as an alternative to traditional oil and gas energy^1^. China possesses substantial shale gas reserves. Organic-rich shales, mainly classified by sedimentary environment, are grouped into continental, marine, and marine-continental transitional shales. They are characterized by multi-layered distribution, diverse genesis types, and complex later modifications^2^. Following North America’s successful shale gas production, China accelerated shale gas development and achieved major breakthroughs in marine shale gas. Confirmed geological reserves accumulated to 2.96 × 10^12^ m^3^ by the end of 2023, accompanied by 6.765 × 10^11^ m^3^ in technically recoverable volumes. Concentrated primarily within the Sichuan Basin and the surrounding areas, these reserves are found in the Weiyuan, Fuling, Luzhou, Changning, Taiyang, Qijiang and Weirong shale gas fields^3^.

Compared to marine shale gas, continental shale gas exhibits a broader distribution and possesses substantial resource potential. It occurs extensively throughout North and Northeast China, the Northwest, Yunnan-Guizhou-Guangxi, Upper Yangtze and Middle/Lower Yangtze-Southeast regions. Despite its wide presence, the exploration and development of continental shale gas remain in its infancy, and industrial gas flow has only been obtained in the Songliao, Ordos, and Sichuan Basins^4^. Although the current exploration level is relatively low, the continental shale gas development is of particular significance for ensuring the sustained growth of China’s shale gas production and is receiving increasing attention^1^. Continental shale distribution and evolution are influenced by various factors, including paleoclimatic conditions, sedimentary facies, paleosalinity, the preservation and provenance conditions of OM. These factors contribute to the distinct characteristics of continental fine-grained sedimentation, including pronounced heterogeneity, frequent transitions in sedimentary facies, and a high proportion of clay minerals within the reservoir layers^5^. Organic-rich shale is primarily formed in saltwater-brackish water lake sedimentary environments, and a few are formed in freshwater lake sedimentary environments^6^. Within the Triassic Yanchang Formation of the Ordos Basin, the shale deposition of the Chang 7 Member occurred under warm and humid paleoclimatic conditions in a freshwater lacustrine environment. The weathering of newly formed volcanic rocks, the deposition of volcanic ash, along with the so-called lag period after volcanic activity, trigger the explosive reproduction of plankton and significantly enhance the reducing properties of water, creating favorable conditions for the enrichment of OM in black shale^7^. Within the Dongpu Depression, Paleogene shales of the third member of the Shahejie Formation developed under cold and arid paleoclimate conditions. The paleowater had high salinity and was a typical brackish water. The high salinity led to a reduction in the contents of O_2_ and CO_2_ in the water, enhancing its reducing property. However, when the salinity is too high, the harsh environment leads to a sharp decline in the number and variety of species, resulting in a decrease in primary productivity. Therefore, high primary productivity, appropriate salinity and appropriate sedimentation rate are conducive to the enrichment of OM^6^.

The research on the gas-bearing capacity of shale identifies gas content and occurrence states as critical indicators for evaluating continental shale gas resource potential^8^. The main forms of shale gas occurrence are free gas and adsorbed gas, with dissolved gas accounting for a very low proportion. The free gas content is predominantly affected by factors such as temperature, pressure, PV, and water saturation^9,10^. In contrast, the adsorbed gas content is determined by two major categories of factors: internal and external factors. Internal factors include the maturity, abundance, and type of OM, rock and mineral composition, gas composition, pore structure, wettability, and pore water. External factors cover temperature and pressure^11,12^. Previous research results show that among inorganic minerals, clay minerals have enhanced adsorption capacity due to the development of micropores and a large SSA. In terms of organic geochemical characteristics, there is a correlation between the OM abundance and adsorption capacity^13^. The higher the degree of development of OM micropores, the larger the PV and SSA, and the stronger the adsorption capacity. From the perspective of pore structure, the SSA and PV provide space for adsorption, and a large number of well-developed micropores help enhance adsorption capacity^14^. In terms of external temperature and pressure conditions, since adsorption is an exothermic physical process, temperature will have an impact on it. The influence of pressure and temperature varies under different pressure conditions. In a low-pressure environment, the effect of pressure is more significant. In a high-pressure environment, the influence of temperature is even more pronounced^15^. However, because of the greater complexity involved in studying continental shale, the study of its reservoir characteristics is still not in—depth and detailed enough. The key factors influencing gas content in continental shale have not been clearly identified. This impedes the comprehension of continental shale gas accumulation mechanisms and enrichment patterns, thereby delaying the progress of its exploration and development.

Continental organic-rich shale mainly occurs in the coal-bearing strata of large depression or fault basins, but it is more widely distributed in moderate- to small-scale continental basins. Extensive studies have been carried out regarding the development of coal and coalbed methane resources in moderate- to small-scale continental basins. Nevertheless, studies focusing on the geological theories and development technologies related to shale gas remain relatively limited. Therefore, by integrating the experimental data obtained from field emission scanning electron microscopy (FESEM), XRD, and LTN_2_A of the Xiahuayuan Formation shale from the Xuanhua Basin in the Yanshan area, and combining it with the in-situ desorption gas results, the reservoir characteristics of continental shale were systematically revealed, and both qualitative and quantitative gas content assessments were carried out. Furthermore, this study thoroughly examined the key factors influencing the content of adsorbed and free gas in continental shale, with the goal of offering theoretical guidance for shale gas exploration within the Xiahuayuan Formation of the region.

## Geological background

Situated in the central-northern sector of the North China Craton, the Yanshan area constitutes part of the Yanliao rift system. This system features a central uplift zone flanked by bilateral depressions. The central uplift comprises the Mihuai and Shanhaiguan Uplifts, formed by Paleoproterozoic metamorphic rocks and multistage granites. The northern depressions include Xuanlong, Jibei, and Liaoxi; the southern depressions encompass Jidong and Jingxi.

**Fig. 1:**
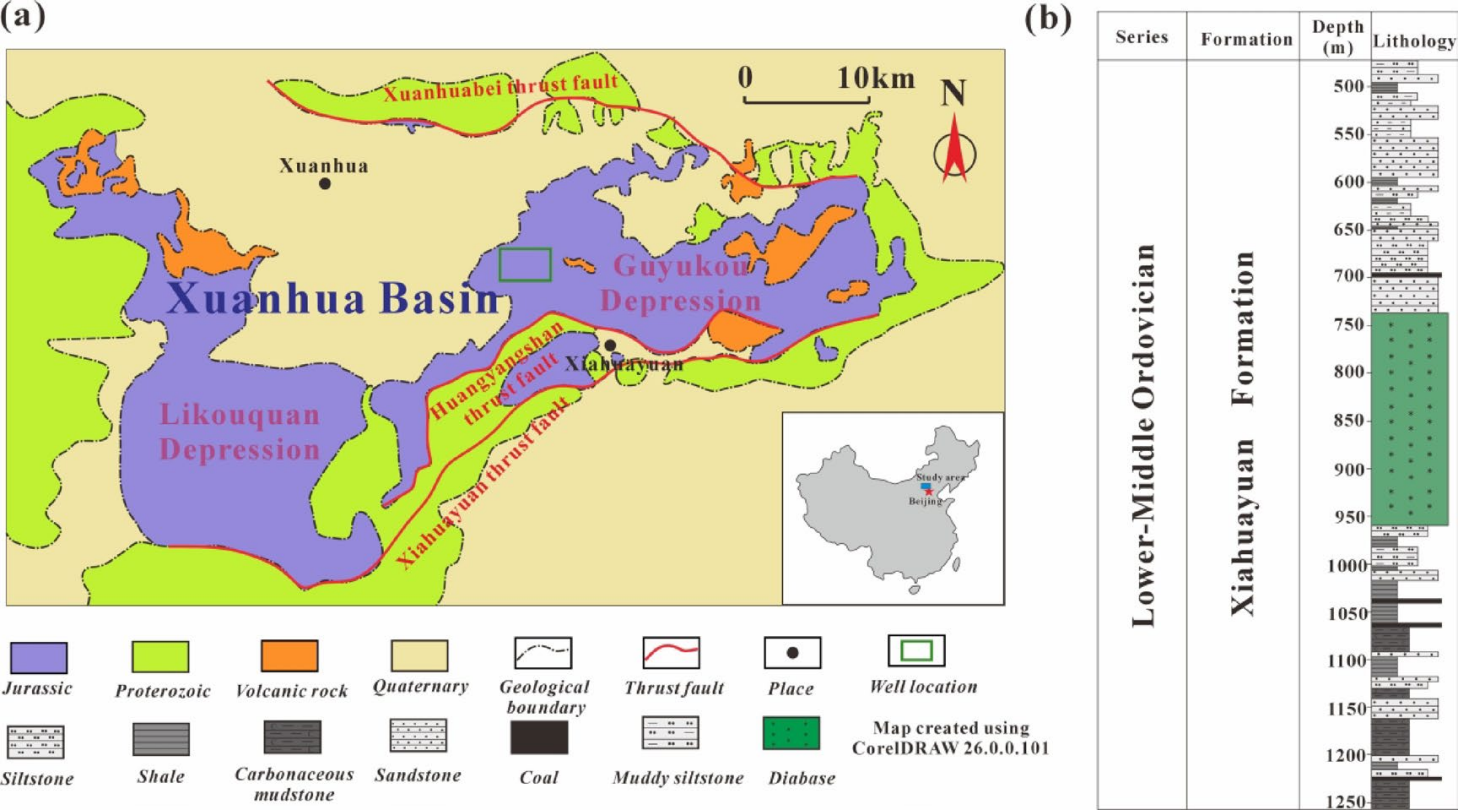
Schematic map of the location of X well in Xuanhua Basin (modified after Tao et al^19^) (**a**) and comprehensive stratigraphic column (**b**). Figures were produced by CorelDRAW 2025 software ( https://www.coreldrawchina.com/).

As a minor flexural basin within the Yanshan fold belt, the Xuanhua Basin occupies the structural junction of the northern segment of the Taihang mountain fold belt and the western segment of the Yanshan fold belt, exhibiting a monocline structure that gently dips toward the south (Fig. 1a). The geological evolution of this basin has been strongly influenced by intense deformation caused by continental collisions and the subduction of plates from different directions, including the closure and collision of the Paleo-Asian Ocean along the Suolon-Linxi suture zone (Permian–Triassic)^16^; the collision and closure of the Mongolia-Okhotsk Ocean (Jurassic-Early Cretaceous)^17^ ; the sequential northwestward subduction of the Izanagi and Pacific plates beneath East Asia^18^; and the Middle Triassic-Jurassic collisional effects from the Qinling–Dabie–Sulu orogen^16^. The basin’s structural framework is primarily influenced by thrust fault systems located on both its northern and southern margins. The Xiahuayuan thrust fault on the south side extends in a NE direction, with the fault plane dipping toward the SSE. The Xuanhua North thrust fault on the north side extends along the EW direction, with a significant northward dip of the fault plane.

The Xuanhua Basin’s basement primarily consists of Archean metamorphic crystalline basement rocks, Proterozoic shallow marine carbonate and clastic rock strata. Mesozoic sedimentary strata progress stratigraphically upward through the Xiahuayuan, Jiulongshan, Tiaojishan, and Tuchengzi Formations. Among them, the Xiahuayuan Formation mainly develops shale, carbonaceous mudstone, coal seams and argillaceous siltstone, etc. The shale development thickness constitutes approximately 20% of the overall thickness of the Xiahuayuan Formation. In addition to clastic sedimentary rocks, diabase intrusions are also widely developed in this stratum, which has significantly influenced the original thickness and thermal evolution process of the surrounding strata (Fig. 1b). The sedimentary facies of the Xiahuayuan Formation are principally characterized by fan delta deposits, supplemented by alluvial fan and lacustrine environments. Lake deposition is manifested as the shore shallow lake deposition, while fan delta deposition is widely distributed vertically, mainly presenting as subfacies deposition in the leading edge and the fan delta plain of the fan delta.

## Sample and experimental methods

Shale samples were obtained from the Lower Jurassic Xiahuanyuan Formation in X Well located in the Xuanhua Basin, with burial depths varying between 506 and 1250 m. 42 shale samples underwent a series of analyses, including TOC content, XRD, vitrinite reflectance (R_o_), FESEM, LTN_2_A, and in-situ desorption experiments.

TOC content determination was carried out using the U.S.-manufactured LECO CS230 carbon–sulfur analyzer. The specific testing method and standard reference were based on “Determination for TOC in sedimentary rock” (GB/T19145-2003). A total of 42 samples were analyzed. First, samples were pulverized to 80 mesh (0.2 mm). Subsequently, subject 0.1 g of the powder to treatment with dilute hydrochloric acid to eliminate carbonates. After that, wash and dry it, and measure the TOC content. The XRD analyses were carried out following the industry standard SY/T5163-2018.13. shale samples were subjected to whole-rock mineralogical characterization using a Bruker AXS D8 Discover X-ray diffractometer. A 40 μm powder sample was placed in the measuring instrument under conditions of 40 mA current, 40 kV voltage, a rotation angle range of 3° to 85°, and an angular rotation rate of 2°/min. The relative mineral composition was then calculated based on the peak areas corresponding to specific reflections in the acquired diffraction pattern. The determination of vitrinite reflectance follows the industry standard SY/T5124-2012. First, 16 shale samples were comminuted to 20–40 mesh. They were then combined with epoxy resin, consolidated, pressed into slices, and polished. Finally, the vitrinite within the shale samples was examined using a 50 × oil immersion objective.

The FESEM observations were conducted in accordance with the “Analytical method of rock sample by SEM” (SY/T5162-2014). Cubic samples (2 cm × 2 cm) underwent initial coarse abrasion with sandpaper. Subsequent argon-ion polishing was performed, which included gold sputter-coating. Final microstructural observation was conducted using a U.S.-manufactured FEI Quanta 250 FEG SEM at an accelerating voltage of 10 kV and a working distance of approximately 9 mm.

The LTN_2_A complies with Chinese national standard GB/T 21650.2-2008. A total of 10 shale samples were analyzed. After being dried and degassed under vacuum at 378 K for over 12 h, the 0.25 mm powder samples underwent N_2_ adsorption analysis at a constant temperature of 77 K. The pore size distribution (PSD), PV, and SSA were calculated based on the adsorption–desorption curve using the NLDFT and BET models.

The gas content of 42 shale samples was directly determined by the in-situ gas-desorption method. According to the desorption steps, the gas content was divided into three parts: desorbed gas, lost gas, and residual gas. The operation process of the desorption method involves accurately recording the time for lowering the drill, raising the drill, and the arrival of the core at the wellhead during the drilling process. Core samples undergo prompt sealing and are placed in desorption vessels after extraction. Through temperature simulation control, the natural desorption gas volume of shale under formation conditions is measured. Following desorption, the loss gas volume is obtained using the USBM method. Finally, the core is crushed, and the residual gas volume is measured.

## Result

### Organic geochemical characteristics

**Fig. 2:**
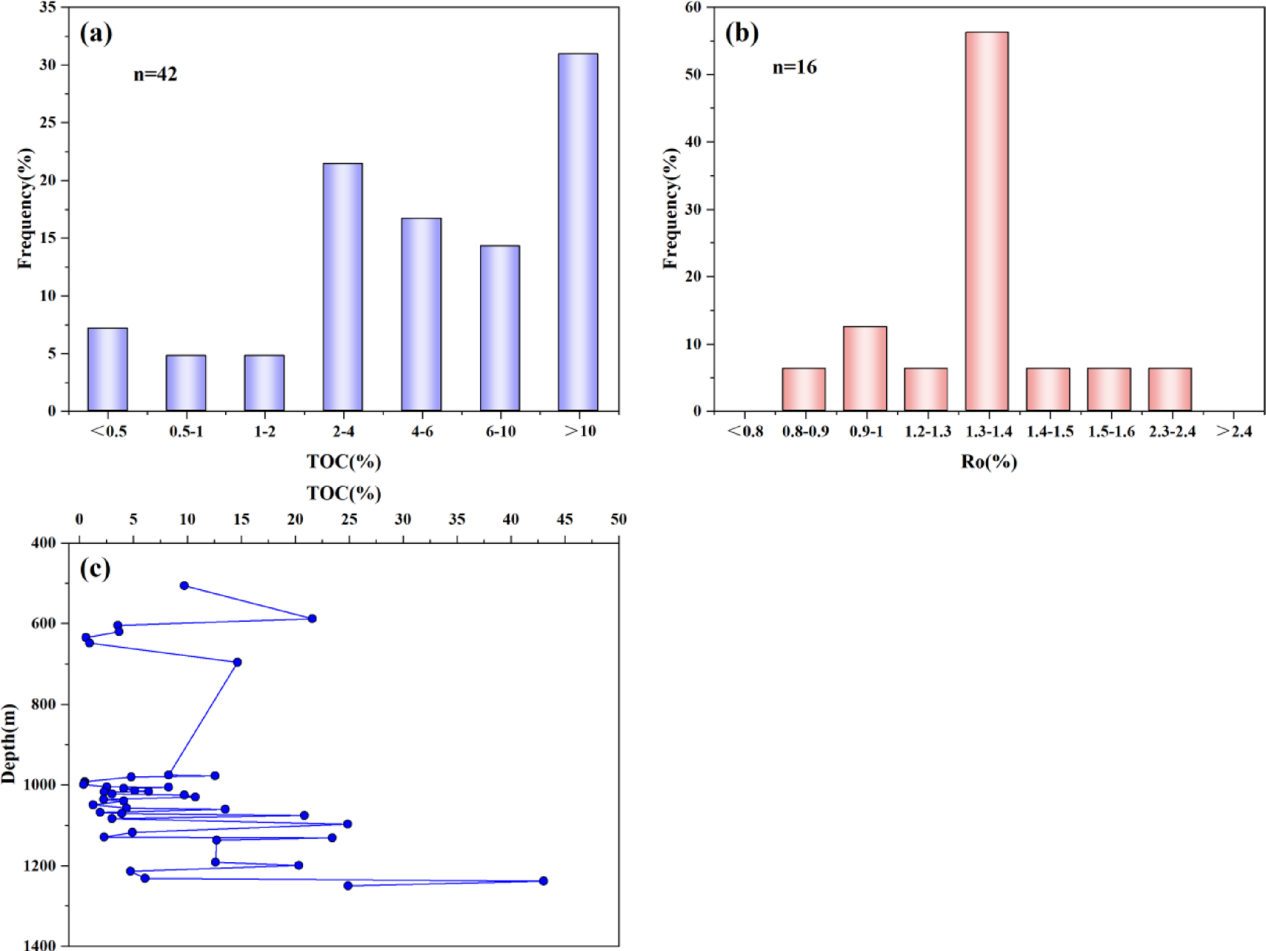
The distribution map of OM characteristics of the Xiahuayuan Formation shale in the Xuanhua Basin, Yanshan area. (**a**) Frequency distribution of TOC content; (**b**) Frequency distribution of R_O_; (**c**) The relationship between TOC and burial depth.

TOC content quantifies the OM abundance, which is the fundamental prerequisite for shale gas generation and a key determinant of hydrocarbon potential^20,21^. Analyses of the Xiahuayuan Formation shale reveal significant TOC heterogeneity (0.4–43%, avg. 8.8%). Among them, 83.3% of the samples have TOC values exceeding 2% (Fig. 2a). The Xiahuayuan Formation shale belongs to carbon-rich shale, with good hydrocarbon generation potential. Moreover, TOC content exhibits a progressive upward trend with increasing burial depth (Fig. 2c). OM maturity, a critical proxy for thermal evolution stage and hydrocarbon generation^22,23^, is indicated by Rₒ values (0.9–2.3%, avg. 1.4%). The R_o_ range indicates maturity to high-maturity conditions, confirming that the Xiahuayuan formation has entered into the main gas window (Fig. 2b).

**Fig. 3:**
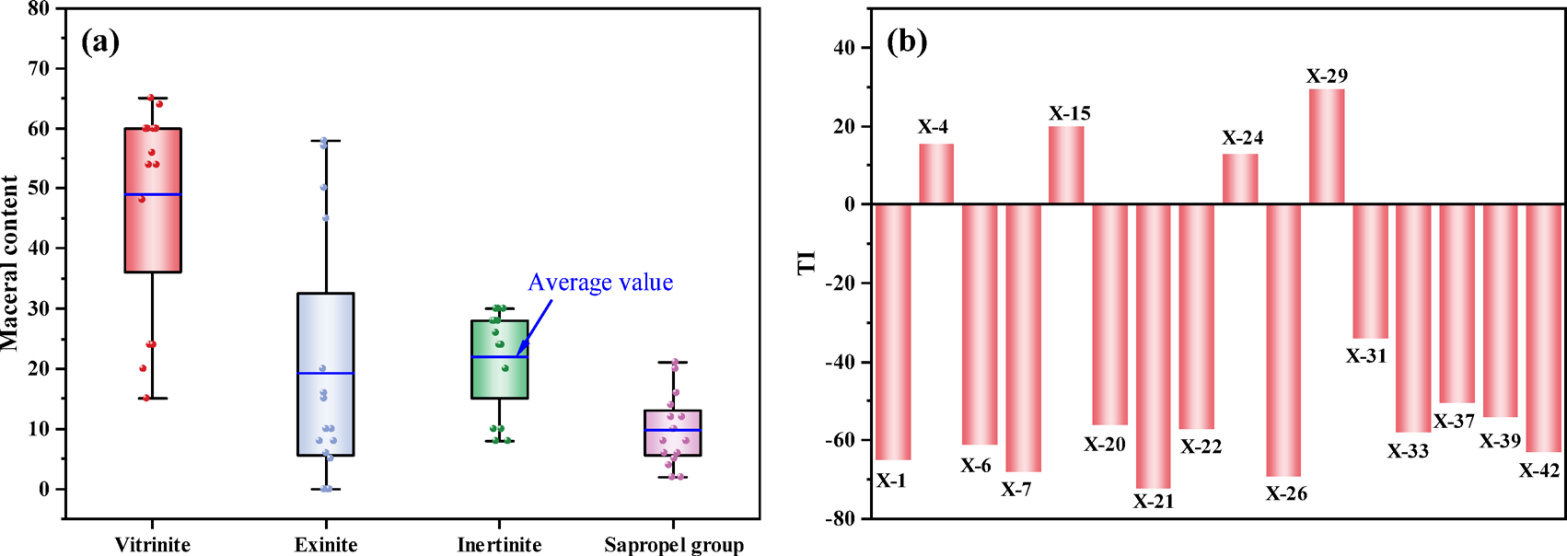
The distribution map of kerogen characteristics of the Xiahuayuan Formation shale in the Xuanhua Basin, Yanshan area. (**a**) Microscopic component; (**b**) type index.

The OM type directly determines the hydrocarbon generation potential, the evolution of hydrocarbon formation processes and the products formed, and also directly affects the content and occurrence state of shale gas^24,25^. TI is the kerogen type index, expressed as follows: TI = (sapropelite × 100%) + (exinite × 50%)-(vitrinite × 75%)- (inertinite × 100%). The results of the kerogen microscopic component content test show that the maceral compositions of kerogen in the Xiahuayuan Formation shale contain predominantly vitrinite (15–65%, avg. 49%) with subordinate inertinite (8–30%, avg. 22%), alongside exinite (0–58%, avg. 19.2%) and sapropelite (2–21%, avg. 9.8%), indicating that the OM mainly originated from terrestrial higher plants (Fig. 3a). According to the calculation results of TI (Fig. 3b), the OM is mainly composed of kerogen type Ⅲ and a minor presence of kerogen type Ⅱ_2_, further indicating that the shale is primarily characterized by gas generation^26^.

### Mineralogical composition characteristics

**Fig. 4:**
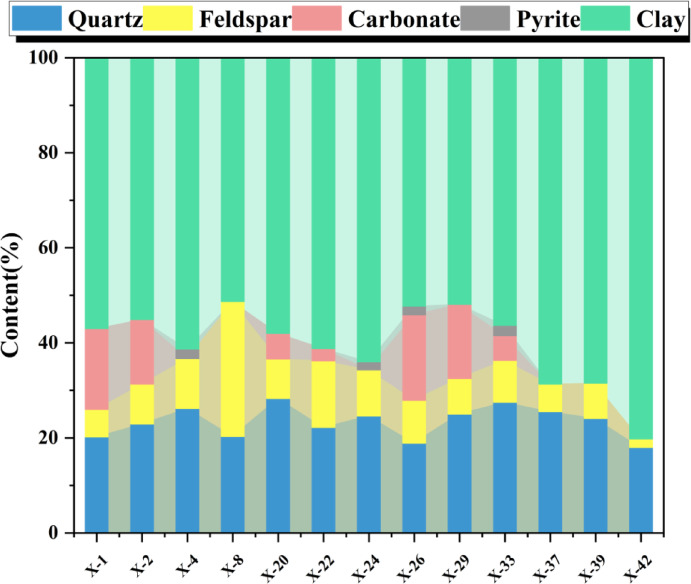
The distribution map of whole-rock mineral composition of the Xiahuayuan Formation shale in the Xuanhua Basin, Yanshan area.

As both gas-generating and gas-bearing layers, the mineral composition and content play a pivotal role in influencing the rock mechanical properties of shale, the development of pores and fractures, and the enrichment and storage of shale gas^13,27^. Whole-rock mineral analysis reveals complex mineral composition in the Xiahuayuan Formation shale of the Xuanhua Basin. Substantial compositional variability among samples demonstrates significant lithological heterogeneity. The main mineral types include clay minerals and quartz. Additionally, minor amounts of feldspar and pyrite can also be observed (Fig. 4). Among them, clay minerals dominate in the shale (51.3–80.2%, avg. 60.4%), substantially exceeding marine shale averages. High content of clay minerals significantly impacts the plasticity and pore structure of shale, thereby affecting gas adsorption and storage capacity^28^. Quartz constitutes 18.0–28.3% (avg. 23.4%). Compared with marine shale^29^, its quartz content is not high, but it can provide certain support for the pores in the shale and is conducive to the preservation of the pores^30,31^. Feldspar (1.8–28.4%, avg. 9.6%) and pyrite (avg. 0.6%) constitute minor fractions.

**Fig. 5:**
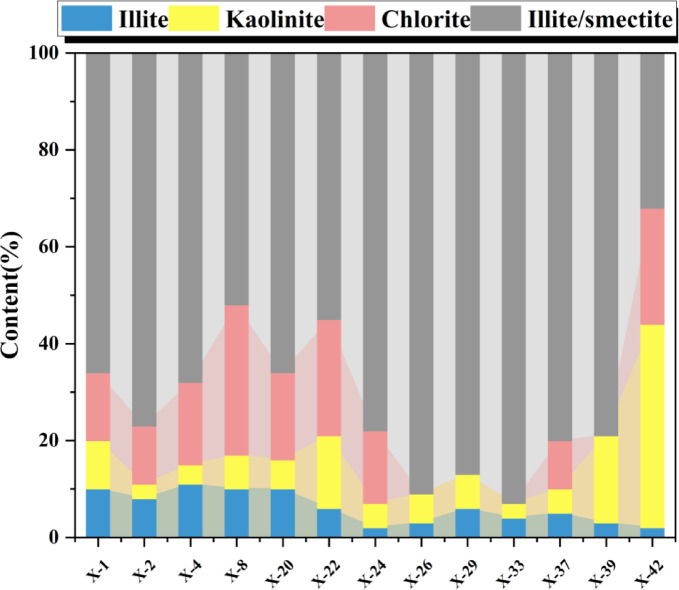
The distribution map of clay mineral composition of the Xiahuayuan Formation shale in the Xuanhua Basin, Yanshan area.

Clay minerals possess catalytic activity conducive to hydrocarbon generation. Meanwhile, the pores developed in clay minerals have a relatively large SSA, which is favorable for the adsorption of shale gas and constitutes an important storage space for shale gas^27,28^. In the shale samples of the Xiahuayuan Formation in the Xuanhua Basin, clay mineral are dominated by illite–smectite (I/S) mixed-layer (32–93%, avg. 71.1%), with subordinate amounts of chlorite (0–31%, avg. 12.7%), kaolinite (3–42%, avg. 10.1% ), and illite (2–11%, avg. 6.2%) (Fig. 5). Compared with marine shale, continental shale has its unique characteristics, which are manifested in a high content of I/S mixed layer and the absence of smectite. This may be related to the slightly acidic sedimentary environment.

**Fig. 6:**
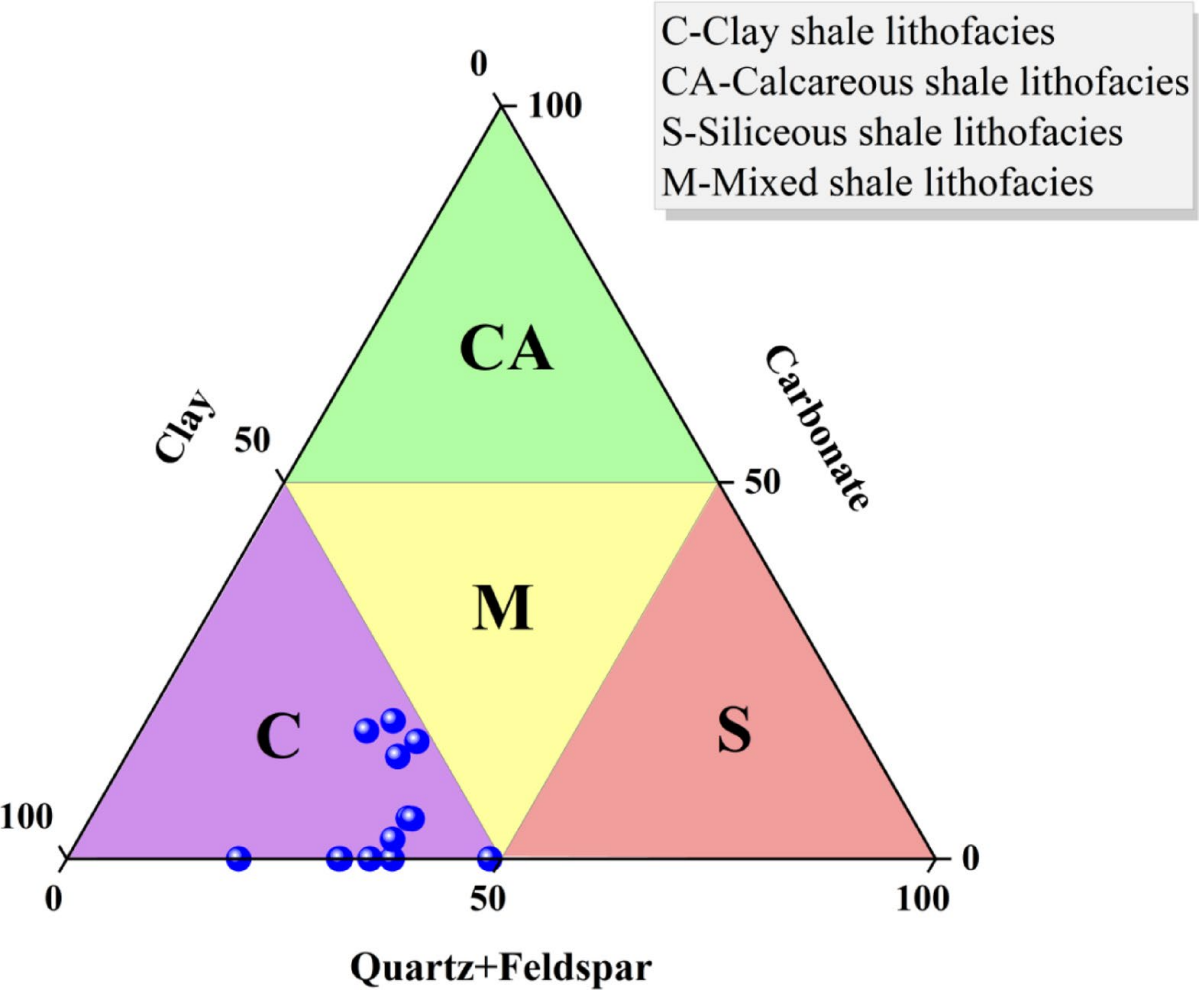
The ternary diagram of shale mineral composition of the Xiahuayuan Formation in the Xuanhua Basin, Yanshan area.

The pore characteristics, mineral composition, and OM abundance of shale are all controlled by the shale lithofacies, which affects shale gas enrichment^32,33^. For the Xiahuayuan Formation shales, the lithofacies categorization utilized ternary diagrams of clay minerals, carbonate minerals and siliceous minerals (quartz + feldspar). A total of four lithofacies associations were delineated, namely calcareous shale, clay shale, siliceous shale, and mixed shale lithofacies. Clay shale lithofacies is the dominant type (Fig. 6).

### Pore Type

The PSD in shale is wide and highly heterogeneous. As the storage site for shale gas, its development characteristics are one of the key indicators for shale gas enrichment^34,35^. According to the characteristics and causes of pore structures, as well as other influencing factors, pores and fractures in shale reservoirs can be primarily categorized into three types: organic pores, inorganic pores, and microfractures. Among these, inorganic pores are further subdivided into intragranular (IntraG) and intergranular (InterG) pores^36^. According to the results of FESEM, the Xiahuayuan Formation shale samples have developed various types of pores, mainly inorganic pores.

**Fig. 7:**
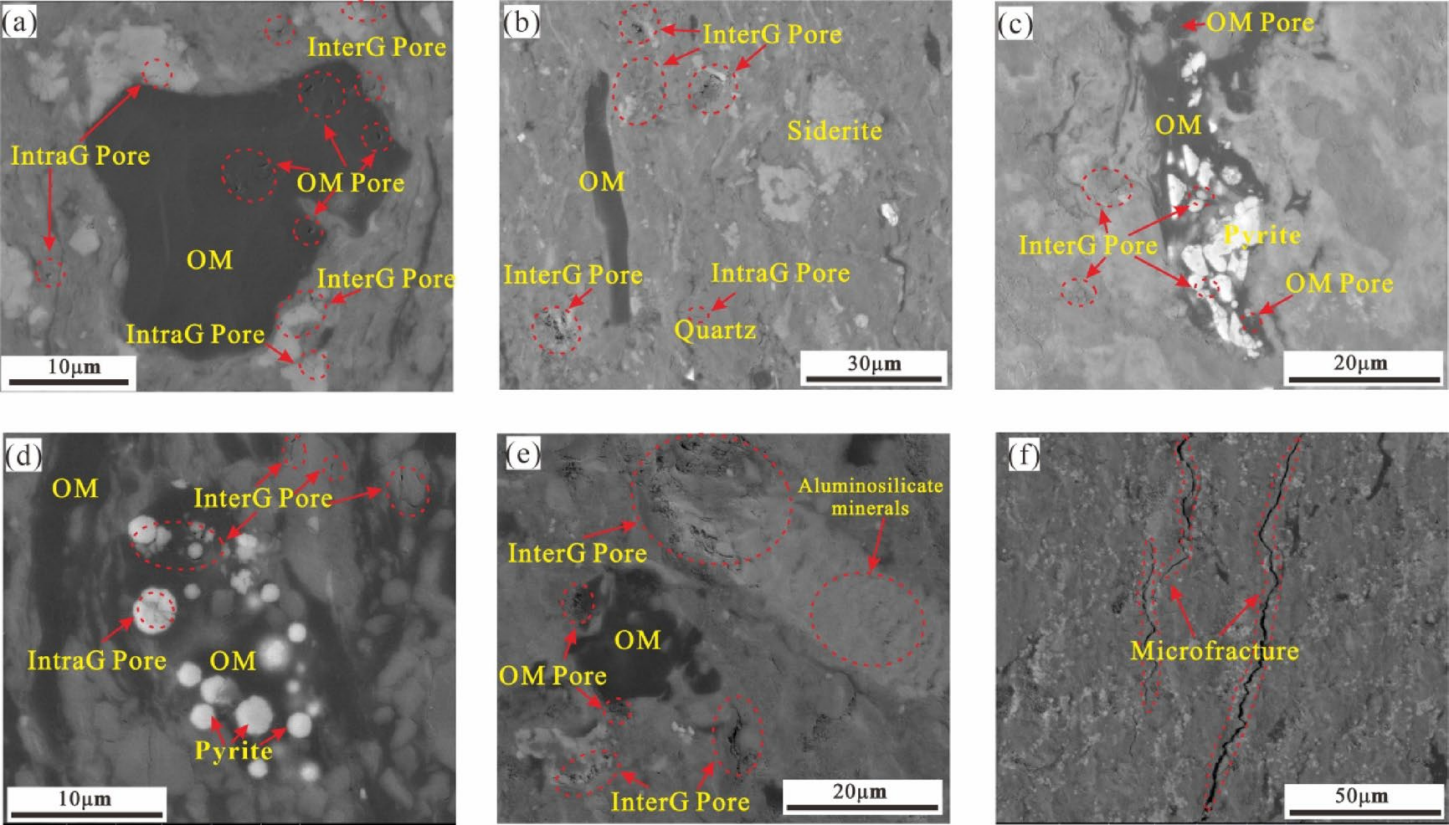
Microscopic Pore Types and Characteristics of the Xiahuayuan Formation Shale in the Xuanhua Basin, Yanshan Area. (**a**) InterG pores, IntraG pores and OM pores in sample X-1; (**b**) interG pores and OM in sample X-4; (**c**) Pyrite, InterG pores and OM pores in sample X-22; (**d**) Pyrite, InterG pores and OM in sample X-31; (**e**) OM pores and InterG pore in sample X-32; (**f**) Microfractures in sample X-29.

OM pores are located within the OM and are mainly controlled by kerogen type, OM abundance, and thermal evolution degree. Morphologically, they are mostly distributed sporadically in a circular or irregular shape, or appear in aggregate forms such as bead-like or honeycomb type, which serve as crucial storage spaces for shale gas. During shale gas exploration and production, the characteristics of OM pores play a crucial role in gas accumulation and migration^37,38^. The FESEM results indicate that the OM within the Xiahuayuan Formation shale predominantly appears in massive or banded forms. The OM pores are poorly developed, mainly presenting as isolated elliptical or slit-shaped pores within the OM. These pores are relatively regular in shape and have smaller diameters, with poor connectivity, and they are the main type of OM pores (Fig. 7a,c,e).

Inorganic pores are those that form within shale reservoirs and are closely associated with inorganic minerals (e.g., feldspar, quartz). The inorganic pores of the Xiahuayuan Formation shale include two types: InterG and IntraG pores. InterG pores are predominantly secondary InterG pores, which develop in the form of slits or irregular angular shapes along the boundaries of brittle mineral or between clay mineral. The pore edges are irregular, with good connectivity. The pore diameters vary widely in scale and are distributed without any regularity(Fig. 7a–e). InterG pores are the main component of inorganic pores, and they are greatly influenced by formation pressure and diagenesis, which play a crucial role in influencing the storage and migration of shale gas^39^. IntraG pores primarily refer to the pores that are distributed in an isolated manner within mineral grains. IntraG pores are occasionally observed in the shale, mainly including dissolution pores formed in minerals (e.g., calcite, feldspar) and pyrite intercrystalline pores. They are elongated or irregular polygons with small pore diameters. The intercrystalline pores of pyrite usually coexist with OM in the form of filling or encapsulation (Fig. 7a,d). Compared with InterG pores, IntraG pores are more scattered in distribution and have poorer connectivity among pores, resulting in a comparatively limited influence on the storage and migration of shale gas.

Microfractures significantly contribute to the enrichment and occurrence of shale gas in continental settings, as well as to its development. They not only provide necessary space for gas storage during the shale gas generation stage process but also serve as connection channels between micropores and between micropores and macrofractures, significantly promoting the effective migration of shale gas^40^. The FESEM results show that the Xiahuayuan Formation shale mainly develops interlayer fractures of clay minerals, which are mostly serrated bending, with uneven widths and a certain degree of continuity (Fig. 7f). These fractures are mainly formed by shrinkage cracks resulting from mineral transformation and dehydration, or are produced by mechanical compaction during diagenesis.

### Pore structure characteristics

**Fig. 8:**
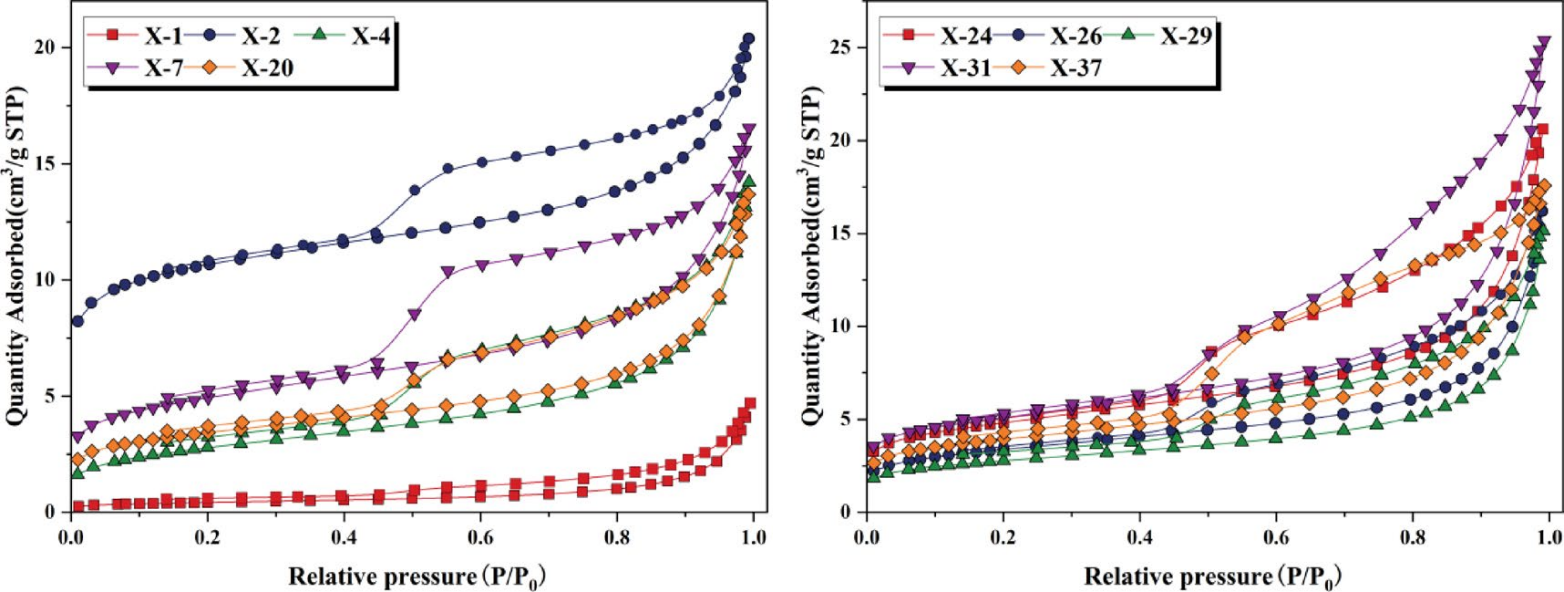
The LTN_2_A curves of shale from the Xiahuayuan Formation in the Xuanhua Basin, Yanshan area.

LTN_2_A experiments can effectively characterize the shale’s pore structure. The results indicate a progressive enhancement of N_2_ adsorption capacity with increasing pressure. In the extremely low-pressure stage where P/P_0_ < 0.01, the adsorption isotherm rises rapidly, corresponding to the filling of micropores, indicating that micropores are well developed. As P/P_0_ increases to 0.45, the growth rate of adsorption capacity slows down, and the adsorption curve shows a gentle upward trend, reflecting the monolayer adsorption process of N_2_ molecules. When P/P_0_ reaches the threshold value of 0.45, as P/P_0_ increases, the monolayer saturation gradually transitions into multilayer adsorption. When P/P_0_ ranges from 0.45 to 0.9, the desorption curve exhibits a desorption hysteresis phenomenon. The desorption and adsorption curves gradually separate, forming a hysteresis loop. When P/P_0_ transitions from 0.9 to 1, the adsorption curve steepens rapidly. As the relative pressure approaches 1.0, the phenomenon of gas molecule condensation occurs, but the adsorption is not yet saturated, indicating that the shale sample contains macropores (Fig. 8). According to the IUPAC classification of LTN_2_A isotherms^41^, the N_2_ adsorption hysteresis loop curve types of the Xiahuayuan Formation shale are similar to H_2_ and H_4_ types, suggesting that the pore morphology of the shale is mainly composed of plate-like slit-shaped, ink-bottle-shaped, and wedge-shaped pores.

**Fig. 9:**
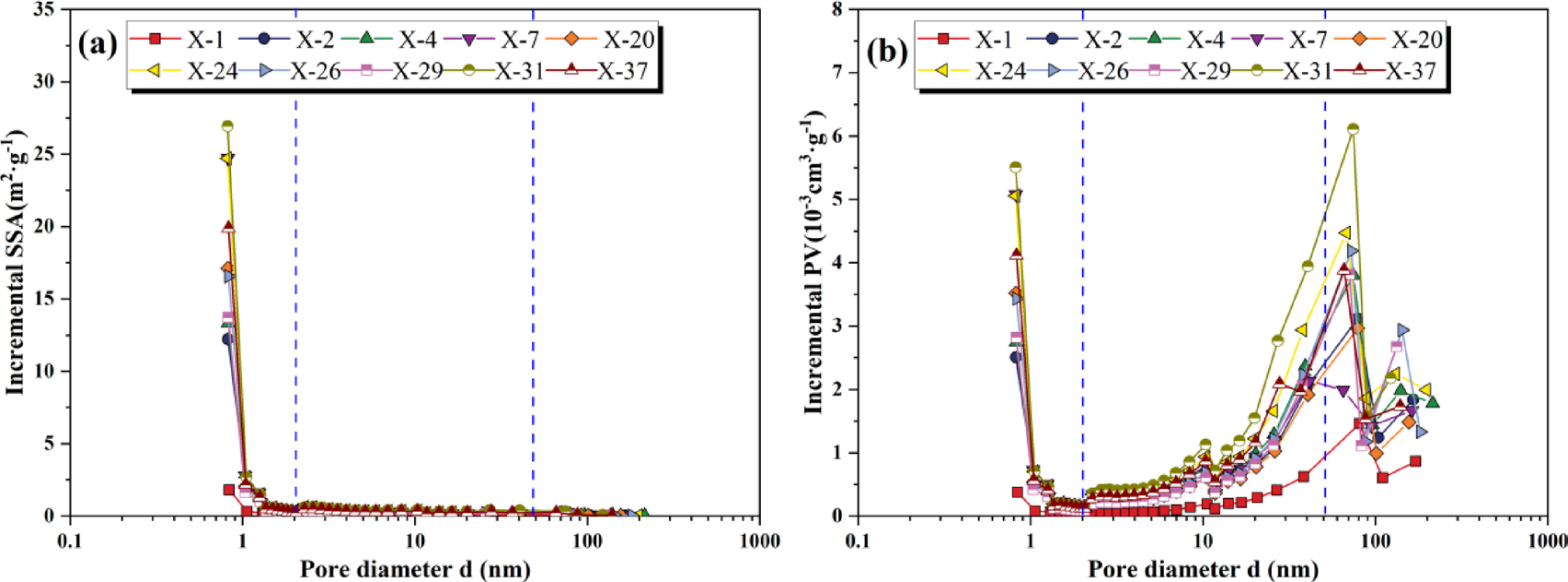
The PSD curve of the Xiahuayuan Formation shale in the Xuanhua Basin, Yanshan area. (**a**) SSA; (**b**) PV.

The pore structure analysis of the Xiahuayuan Formation shale reveals micropores volumes spanning 0.00064–0.0077 cm^3^/g (avg. 0.0052 cm^3^/g) with SSA of 2.59–33.67 m^2^/g (avg. 22.32 m^2^/g). Mesopores exhibit PV between 0.0028–0.018 cm^3^/g (avg. 0.011 cm^3^/g) and SSA of 1.03–7.89 m^2^/g (avg. 5.17 m^2^/g), while macropores range from 0.0029–0.011 cm^3^/g (avg. 0.0073 cm^3^/g) in volume and 0.11–0.47 m^2^/g (avg. 0.32 m^2^/g) in SSA (Fig. 9). It can be known from the PSD curves that the SSA distribution of shale exhibits a unimodal distribution, with the dominant pore size peaking near 1 nm, indicating that the SSA of shale mainly comes from micropores (Fig. 9a). The PV distribution mainly shows a bimodal distribution, with peak pore diameters around 1 nm and 60 nm. The distribution characteristics of different samples are quite similar, indicating that the micropores in the shale are well developed (Fig. 9b).

### Gas bearing characteristics

**Fig. 10:**
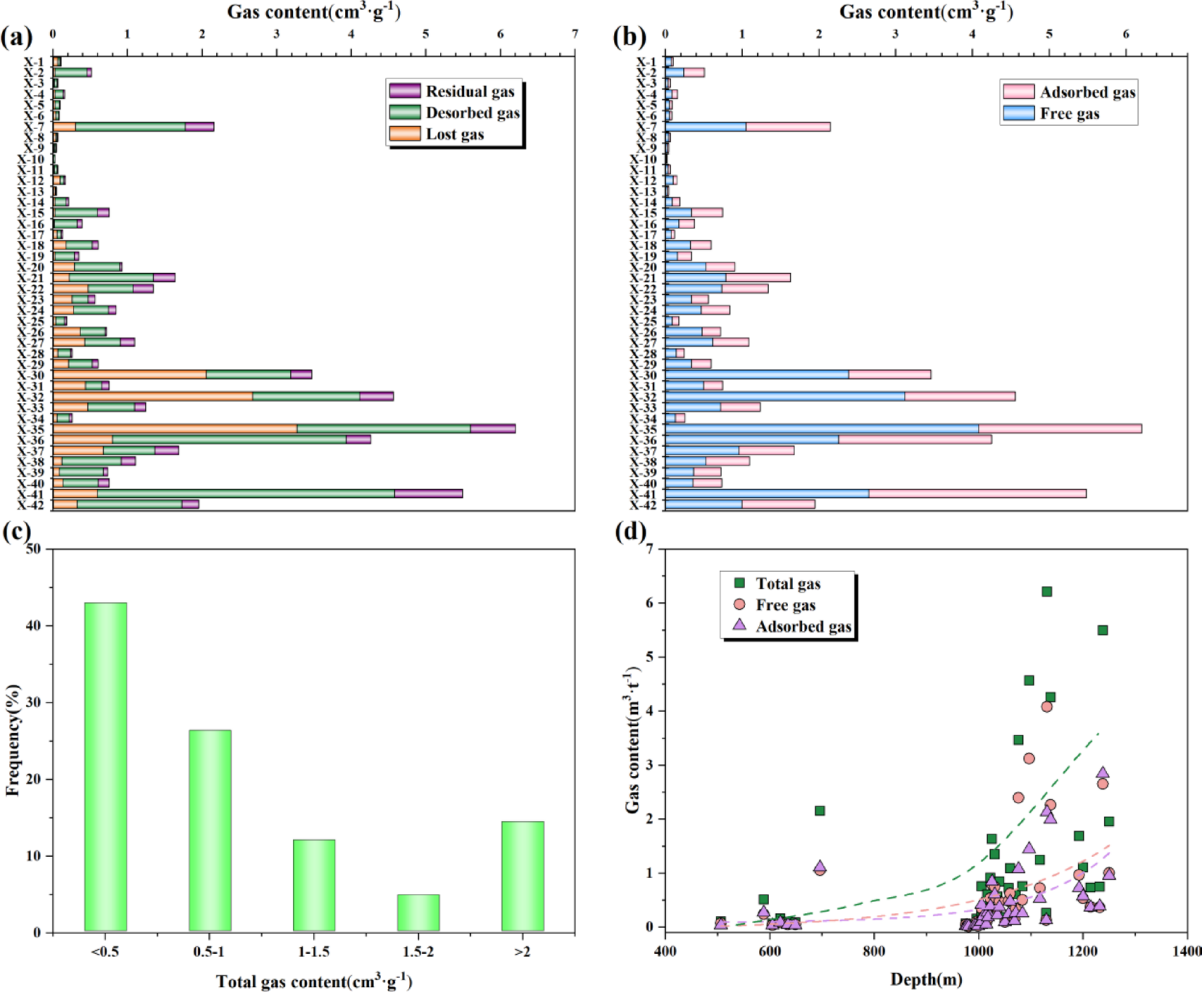
The distribution map of gas-bearing characteristics of the Xiahuayuan Formation shale in the Xuanhua Basin, Yanshan area. (**a**) On-site desorption gas content; (**b**) Adsorbed gas and free gas content; (**c**) Frequency of gas content statistics; (**d**) The relationship between gas content and burial depth.

The determination results of gas content in the shale samples from X well show that the desorbed gas volumes spanning 0.01–4.58 m^3^/t (avg. 0.78 m^3^/t). Lost gas measurements range between 0–3.27 m^3^/t (avg. 0.46 m^3^/t), while residual gas varies from 0–0.91 m^3^/t (avg. 0.15 m^3^/t), yielding total gas contents of 0.02–7 m^3^/t (avg. 1.39 m^3^/t) (Fig. 10a). The total gas content in the deep part is higher than that in the shallow part. A significant portion of the total gas content is attributed to the desorbed gas volume, whereas the residual gas volume constitutes a relatively small fraction. Among them, the gas content less than 0.5 m^3^/t accounts for 42.9% of the total. Gas content between 0.5 m^3^/t and 1.0 m^3^/t accounts for 26.2% of the total. Gas content between 1.0 m^3^/t and 1.5 m^3^/t accounts for 11.9%, while the range from 1.5 to 2.0 m^3^/t accounts for 4.8% of the total. The gas content greater than 2.0 m^3^/t accounts for 14.3% of the total (Fig. 10c). From the perspective of gas content in shale, the Xiahuayuan Formation shale has relatively high gas content and exploration potential.

Based on the in-situ desorption process and principle, establish the relationship between desorption gas content and the state of gas occurrence. The three-segment method was employed to calculate the shale gas ratio at various stages, thereby determining the relative ratio of free gas to adsorbed gas^42^. The corresponding calculation equation is as follows:

1 $${\text{Z}} = \frac{{{\text{x}}_{{\text{l}}} + {\text{x}}_{{\text{d}}} }}{{{\text{x}}_{{\text{d}}} + {\text{x}}_{{\text{r}}} }}$$

where Z represents the ratio of free to adsorbed gas; x_l_ is the lost gas content, m^3^/t; x_d_ is the desorbed gas content, m^3^/t; x_r_ is the residual gas content, m^3^/t.

The calculation results indicate that the free gas content varies between 0.01 and 4.08 m^3^/t (avg. 0.63 m^3^/t); the adsorbed gas content falls within the range of 0.01–2.84 m^3^/t (avg. 0.47 m^3^/t); the ratio of free to adsorbed gas ranges from 0.81 to 2.25 (avg. 1.29) (Fig. 10b). It can be concluded that the Xiahuayuan Formation shale predominantly contains free gas, while adsorbed gas exists in comparatively smaller quantities. Consistent with the variation trend of the total gas content in relation to depth, both free and adsorbed gas contents exhibit a gradual increase as burial depth increases. However the distribution of the overall gas content shows a strong heterogeneity (Fig. 10d).

## Discussion

### The influence of organic geochemical characteristics on gas content

**Fig. 11:**
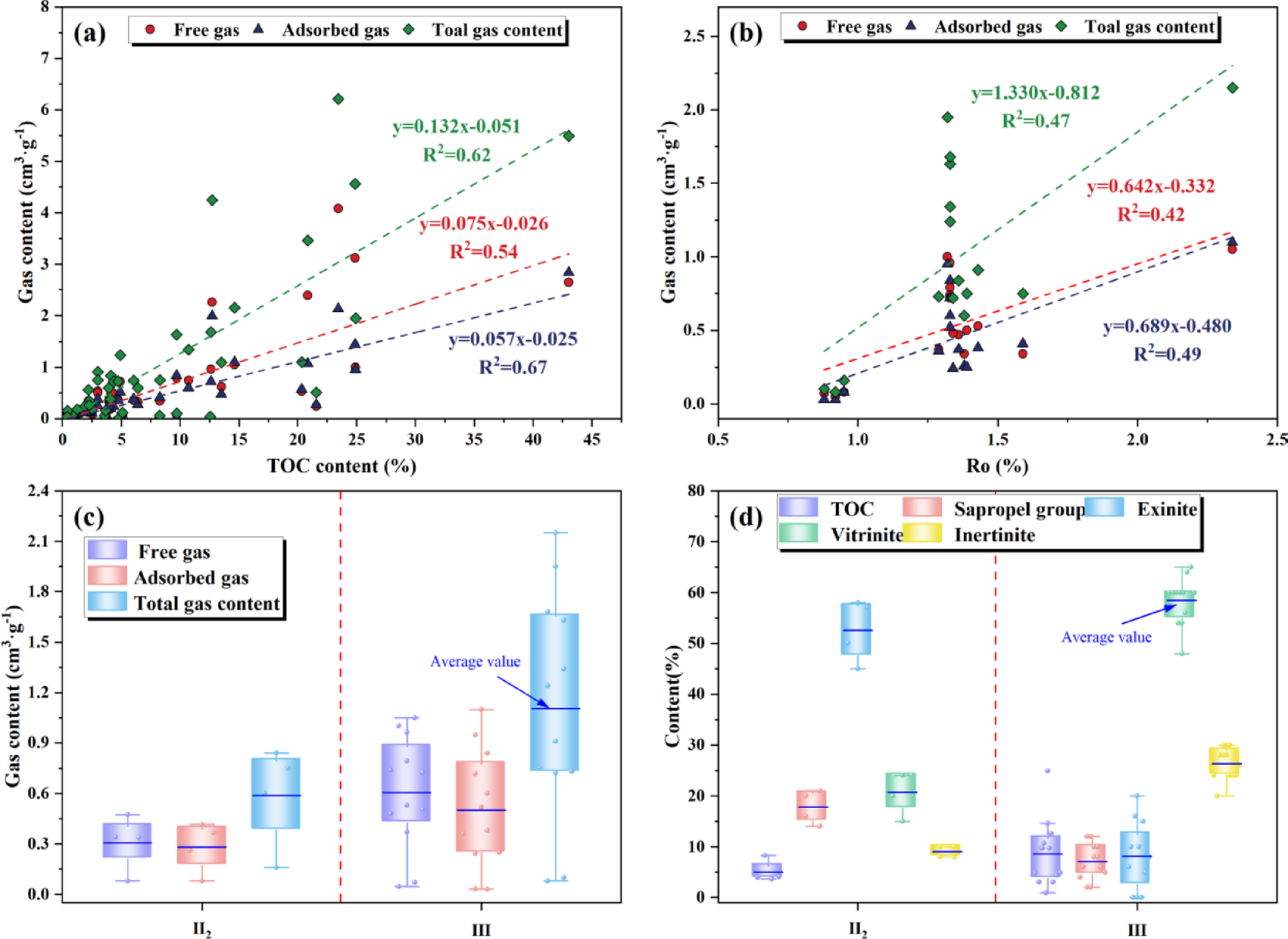
The relationship between shale geochemical parameters and gas content. (**a**) TOC content and gas content; (**b**) R_o_ and Gas content; (**c**) OM types and gas content; (**d**) Microscopic components, TOC content and OM types.

The gas content and TOC content of the Xiahuayuan Formation shale show a relatively obvious positive correlation (Fig. 11a), indicating that as the TOC content increases, so do the adsorbed gas, free gas, and total gas content within the shale. Among them, the adsorbed gas content shows a significant positive correlation with the TOC content. Additionally, the correlation between TOC and both the total gas content and the adsorbed gas content is significantly stronger than that with the free gas content. This phenomenon is mainly attributed to the continuous generation of pores by OM during thermal evolution, especially the micro-nano pores formed by thermal cracking and hydrocarbon generation, which create favorable conditions for the occurrence of shale gas^33,43^. The gas initially produced will first be adsorbed onto the OM pore surface during the the hydrocarbon generation process in shale. When the gas adsorbed on the OM pore surface reaches saturation, the excess gas, driven by pressure difference, is transformed into free gas through seepage and migrates to other pores for storage^16,17^. This process not only increases the porosity of the shale reservoir but also provides ample space for the accumulation of free gas. The higher the OM content, the greater the porosity and the larger the free gas content. Meanwhile, OM exhibits considerable adsorption potential as the material basis for shale gas generation^44^.

In the Xiahuayuan Formation shale, the total gas content in type III OM shale spans from 0.08 m^3^/t to 2.15 m^3^/t (avg. 1.11 m^3^/t); The adsorbed gas content ranges from 0.031 m^3^/t to 1.10 m^3^/t (avg. 0.50 m^3^/t); the free gas content varies between 0.047 m^3^/t and 1.05 m^3^/t (avg. 0.61 m^3^/t) (Fig. 11c). Among them, the total gas, adsorbed gas, and free gas content in type Ⅲ OM shale are all higher than those in type Ⅱ_2_ OM shale. This is mainly attributed to the higher TOC content in Type III OM shale (Fig. 11d). Meanwhile, the difference in OM types results in variations in microscopic components, which leads to distinct gas generation volume, generation mode, generation threshold, and composition among different kerogens^28^. Specifically, the adsorption capacity of different microscopic components varies significantly, with vitrinite > inertinite > exinite. Type Ⅲ kerogen contains a high proportion of vitrinite, which possesses a well-developed aromatic structure (Fig. 11d). This characteristic significantly improves its interaction with CH_4_ molecules, leading to a comparatively higher CH_4_ adsorption capacity. In addition, in type Ⅲ shale, inorganic pores are mainly developed, providing a certain storage space for free gas^45,46^. Compared with type II kerogen, type III kerogen predominantly generates natural gas. Under specific geological conditions, its gas generation cycle may be prolonged, and it exhibits substantial gas generation potential^47^.

OM maturity is one of the key parameters in the evaluation of source rocks, and vitrinite reflectance is an important indicator for measuring OM maturity. At different thermal evolution stages, the hydrocarbon generation and product characteristics of OM are different, which not only affect the hydrocarbon generation of shale, but also influence the gas occurrence state, migration degree and accumulation sites after hydrocarbon generation^45,48^. Through analyzing the relationship between R_o_ and gas content (Fig. 11b), it has been determined that the OM maturity in the Xiahuayuan Formation shale demonstrates a positive correlation with total gas content. Similarly, a positive correlation is observed with adsorbed gas content, as well as with free gas content. The research results show that with the increasing thermal maturity of OM, its capacity to adsorb hydrocarbon gases as well as the capacity to generate free gases is significantly improved. This trend can be attributed to the enhanced gas-generating capability of the shale as OM maturity increases. During the thermal cracking and gas generation process, numerous micro-nano pores develop within the shale reservoir. This significantly enhances both the porosity and SSA, thereby creating more ample space for the storage and accommodation of both free and adsorbed gas^43,49,50^.

### The influence of mineral composition on gas content

**Fig. 12:**
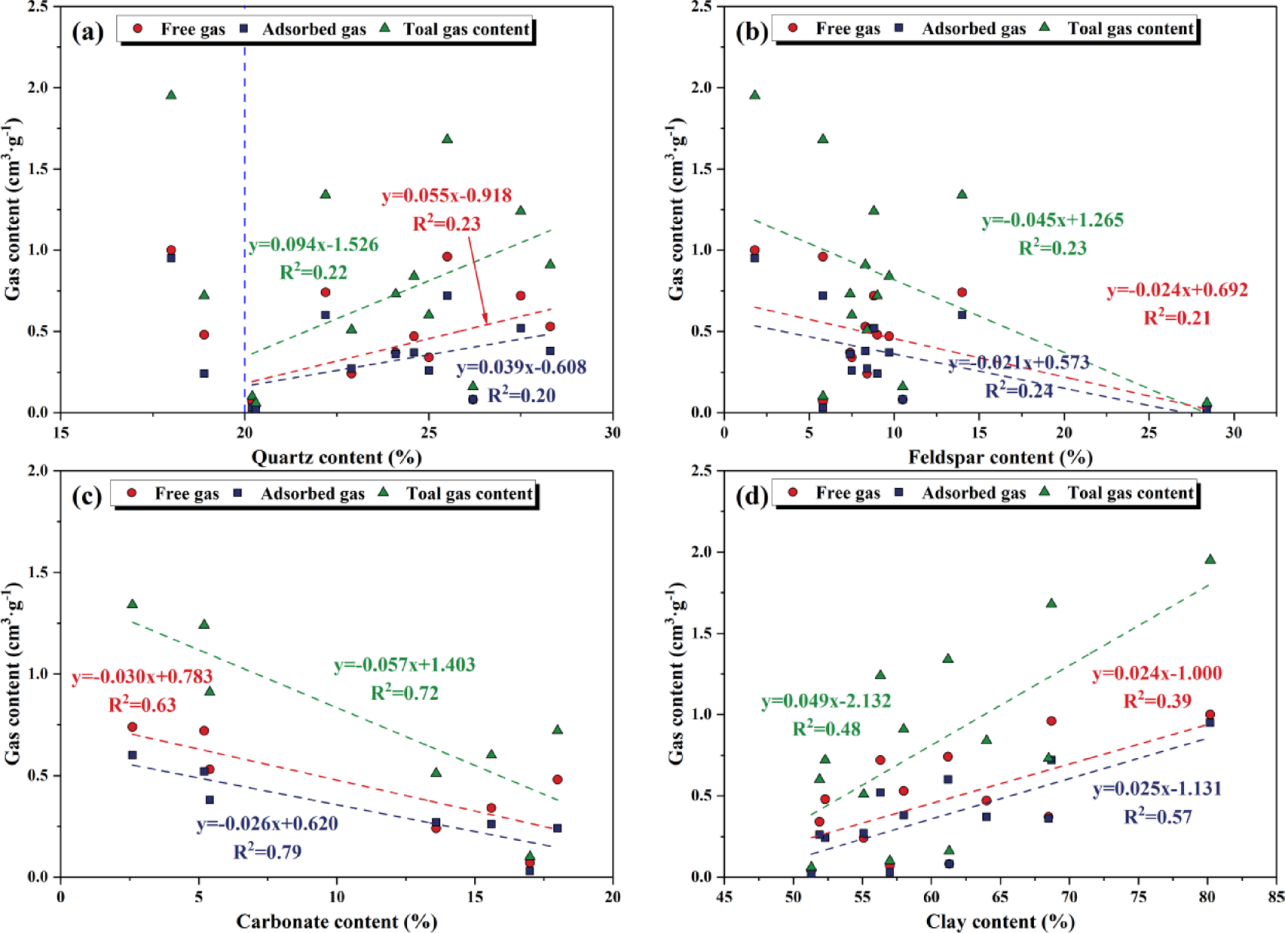
The relationship between the mineral composition of the whole rock and the gas content. (**a**) Quartz content and gas content; (**b**) Feldspar content and gas content; (**c**) Carbonate content and gas content; (**d**) Clay content and gas content.

The mineral composition and its content are key factors influencing play a crucial role in determining the gas content of continental shale and evaluating the reservoir quality. The quartz content of the Xiahuayuan Formation shale does not show a clear relationship with various gas contents (Fig. 12a). However, when the quartz content reaches or exceeds 20.0%, a weak positive relationship exists between various gas contents and quartz content. The main reason is that quartz, as a framework mineral, can form InterG pores and thus becomes an indispensable storage space for shale gas. Furthermore, quartz, as a rigidity grain, is relatively stable in chemical properties. During the burial process of shale, it exhibits significant resistance to compaction and plays a supporting role, effectively reducing the effective stress borne by OM and clay mineral particles, thereby helping to protect OM pores and clay mineral pores from collapse^30,31^. Furthermore, quartz can be classified into detrital quartz and microcrystalline quartz. The larger grains of quartz provide space for the presence of free gas, while the smaller grains of microcrystalline quartz can fill the original InterG pores, increasing the volume of micropores and SSA, and thereby enhancing the number of adsorption sites^51^. Therefore, an increase in the quartz mass fraction leads to a higher content of both adsorbed and free gas.

The feldspar mineral content of shale shows a week negative correlation with total gas content, adsorbed gas, and free gas, which inhibits the gas-bearing capacity to a certain extent (Fig. 12b). Feldspar may occupy pore space through cementation in the later stage of rock formation. Its weak adsorption capacity for CH_4_ and its occupation of some adsorption sites on its surface may affect the accumulation of adsorbed and free gas^52^. Additionally, the carbonate mineral content is comparatively low and exhibits a strong negative correlation with total gas, adsorbed gas, and free gas content (Fig. 12c). The main reason is that carbonate minerals fill the original InterG pores or fractures, and dissolution pores can hardly be developed in the Xiahuayuan Formation shale, which results in a reduction in effective porosity and reduces the storage space for free gas. Meanwhile, carbonate minerals may occupy some adsorption sites, resulting in a reduction in the amount of adsorbed gas^53^. Therefore, an increase in the mass fraction of carbonate mineral leads to a decrease in the content of both adsorbed and free gas.

The clay mineral content of shale shows a positive relationship with total gas, adsorbed gas, and free gas content (Fig. 12d). The main reason is that clay minerals within shale mostly are found as stable OM clay complexes^54^, which is conducive to catalyzing OM during hydrocarbon generation and subsequently forming OM pores, thus having a positive influence on gas content. The Xiahuayuan Formation shale exhibits a relatively high clay mineral content. During the diagenesis process, a significant amount of secondary pores developed, providing storage space for free gas. Meanwhile, clay minerals adsorb hydrocarbon gases through van der Waals forces. Secondary pores significantly increase the SSA of the pores, enhancing the storage capacity of adsorbed gas^28^. In addition, clay minerals are prone to dehydration, which causes internal stress changes and leads to the formation of shrinkage cracks. Therefore, with the increase in the content of clay minerals, various gas contents show an increasing trend.

**Fig. 13:**
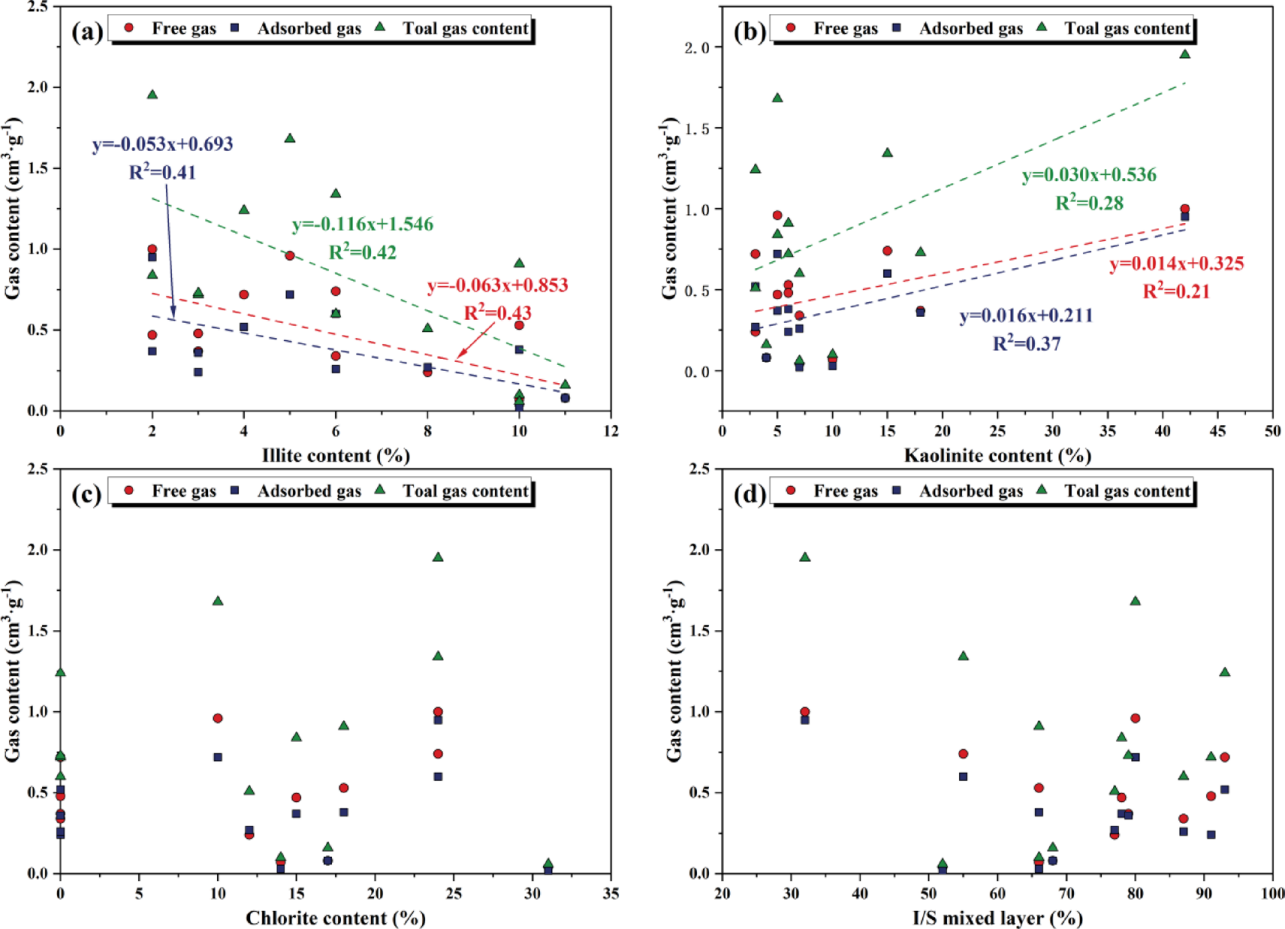
The relationship between clay mineral composition and gas content. (**a**) Illite content and gas content; (**b**) Kaolinite content and gas content; (**c**) Chlorite content and gas content; (**d**) I/S mixed layer and gas content.

The illite content in clay minerals shows a negative relationship with total gas, adsorbed gas, and free gas content (Fig. 13a); the kaolinite content is positively correlated with total gas, adsorbed gas, and free gas content (Fig. 13b); while chlorite and I/S mixed layers have no obvious correlation with each gas content, and their contributions can be ignored (Fig. 13c,d). The main reason is that illite may occupy the original pores or fractures through cementation in the later stage of diagenesis. Illite has a stable interlayer structure, with underdeveloped internal pores and fewer gas adsorption sites. Meanwhile, the surface charge density of illite is relatively low, and the adsorption energy is small, resulting in a weak adsorption effect on gas molecules^27^. Therefore, as the illite content increases, the shale gas content shows a decreasing trend. Kaolinite usually presents a single-layer platy structure, which is easy to form interlayer pores. This provides favorable conditions for serving as a shale gas storage space and promotes gas molecule diffusion. In addition, kaolinite has a high density of surface hydroxyl groups, which mainly interact with the dipole moment of CH₄ molecules through hydrogen bonds, increasing the effective adsorption sites and thereby significantly enhancing its adsorption capacity^24^. Therefore, as the kaolinite content increases, the shale gas content shows an increasing trend. The proportion of smectite layer in the I/S mixed layers is the key factor determining their physical and chemical properties. During the process of buried diagenesis, the smectite layer gradually transforms into the illite layer through the fixation of K^+^ and the substitution of Si and Al. The Xiahuayuan Formation is at a relatively mature stage. The proportion of smectite layers is generally low. The interlayer structure collapse and close due to K^+^ fixation, and the SSA drops sharply, resulting in a lack of significant correlation between the I/S mixed layers and gas bearing.

### The influence of pore structure characteristics on gas content

The microscopic pore structure of shale is closely related to both adsorbed and free gas. The adsorbed gas content primarily depends on the SSA of the pores, while the free gas content is primarily influenced by the PV. The content of adsorbed and free gas is significantly influenced by the radius of the pore throat^51^.

**Fig. 14:**
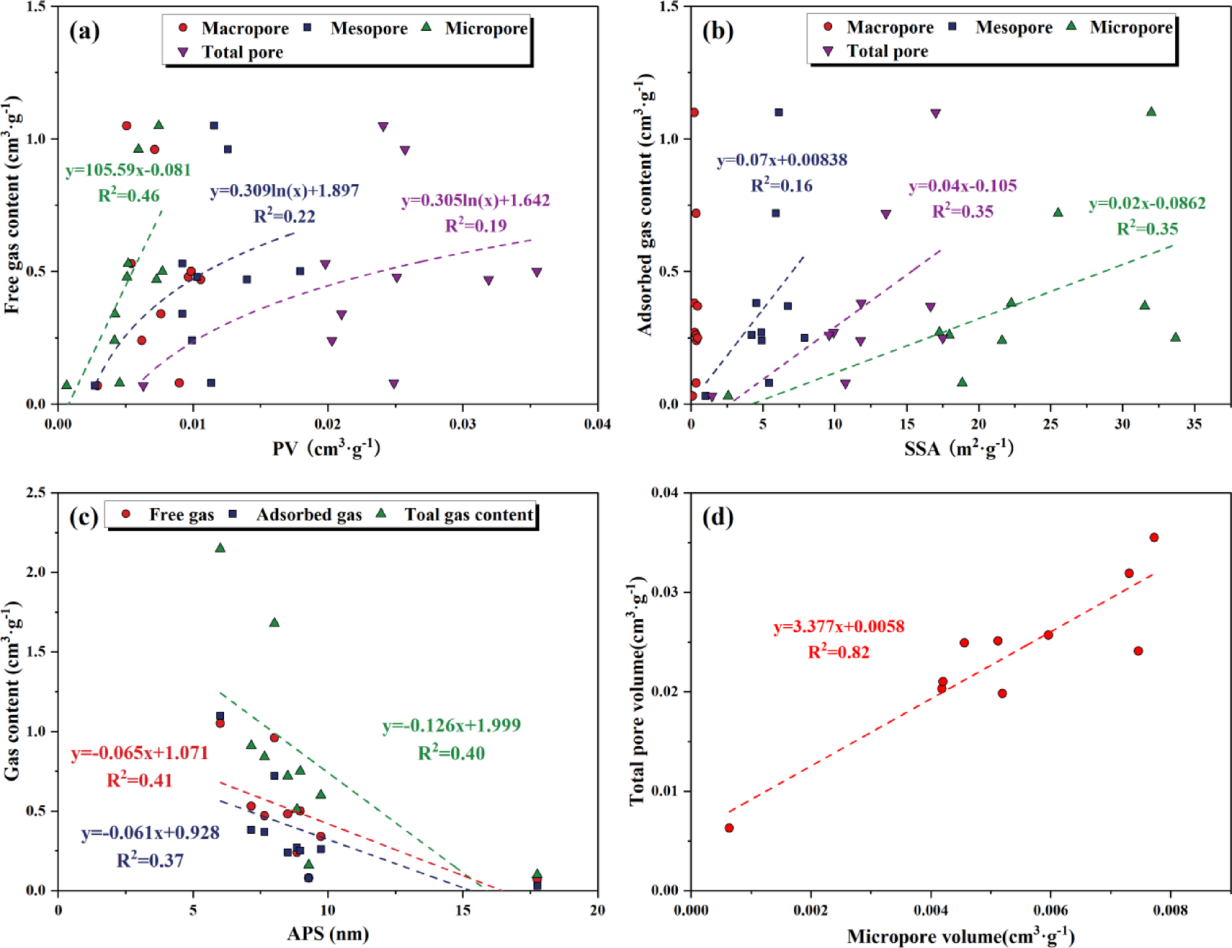
The relationship between pore structure characteristics and gas content. (**a**) PV and free gas content; (**b**) SSA and adsorbed gas content; (**c**) APS and gas content; (**d**) Micropore volume and total PV.

The free gas content of the Xiahuayuan Formation shale in the Xuanhua Basin shows a weak positive correlation with the total PV. Specifically, the micropore volume has a relatively significant positive correlation with the free gas content; the mesopore volume also shows a weak positive correlation with the free gas content. However, no obvious positive correlation is observed between the free gas content and the macropore volume (Fig. 14a). As the pore size of shale decreases, the relationship between the pore size and the free gas content becomes increasingly correlated. The main reason is that CH_4_ molecules usually exist in the form of single or double layer adsorption in micropores. Although the pore diameters of mesopores and micropores are relatively small and the provided PV is limited, the mesopores and micropores, which are mainly composed of InterG pores between minerals in the shale, are relatively well developed, contributing significantly to the total PV. With the increase in micropore volume, the total PV shows an increasing trend, resulting in proportionally greater free gas storage potential (Fig. 14d). Moreover, as the reservoir pressure decreases, the adsorbed gas in the micropores can be converted into free gas during the development of shale gas, and this process may indirectly increase the free gas content.

The adsorbed gas content of the Xiahuayuan Formation shale generally shows a strong positive relationship with the SSA. Specifically, the SSA of micropores demonstrates the strongest positive correlation with adsorbed gas content, while the SSA of mesopores shows a weaker positive correlation. In contrast, the SSA of macropores does not display a notable correlation with the adsorbed gas content (Fig. 14b). With the increase in the pore size of shale, the relationship between SSA and adsorbed gas content gradually weakens. The main reason is that the distance between the pore walls of micropores is smaller, leading to an overlap of the van der Waals force fields acting on methane molecules. Therefore, a stronger adsorption potential is generated within smaller pores^55^. The micropores developed in the shale have a relatively large SSA, providing a greater number of adsorption sites for CH_4_ molecule. As a result, they play a significant role in controlling the adsorbed gas content. However, the macropores do not significantly influence the adsorbed gas content of shale because of their limited SSA^56^.

The APS of the Xiahuayuan Formation shale is negatively correlated with free gas, adsorbed gas, and total gas content, respectively (Fig. 14c). The results show that with the increase in APS, the proportion of micropores decreases, leading to a significant reduction in SSA and a decrease in CH_4_ molecule adsorption sites, which in turn reduces the adsorption capacity of shale. Meanwhile, the reduction in the proportion of micropores results in a notable decline in the total PV of shale, significantly reducing the space for gas storage and making it unfavorable for the free gas storage. Therefore, the simultaneous decrease in both free and adsorbed gas results in a downward trend in the total gas content.

## Conclusion

1. The Xiahuayuan Formation shale in the Xuanhua Basin has a high TOC content and is at a mature to high-mature stage of thermal evolution. The main mineral composition consists of quartz and clay minerals, along with minor amounts of pyrite and feldspar. These characteristics provide the necessary material foundation for the shale gas generation.
2. The pore types of the Xiahuayuan Formation shale are diverse and can be categorized as microfractures, organic pores, and inorganic pores. Well-developed inorganic pores constitute the dominant pore type. The PV distribution shows a bimodal state, mainly consisting of micropores around 1 nm and macropores around 60 nm, indicating that the micropores are relatively developed. The SSA distribution of shale exhibits a unimodal distribution, with the dominant pore size peaking near 1 nm, suggesting that micropores contribute the most to the SSA.
3. The Xiahuayuan Formation shale contains predominantly free gas. As burial depth increases, both free and adsorbed gas contents in the shale show a significant upward trend. It indicates that deep shale may have higher industrial exploitation value. TOC content directly affects the shale gas content, and areas with high TOC content are often the preferred targets for shale gas enrichment. The volume of micropores is positively correlated with the free gas content, while the SSA of micropores is strongly and positively correlated with the adsorbed gas content. However, the APS is negatively correlated with various gas contents. The impact of mineral composition on gas content is rather complex. Clay minerals show a strong positive correlation with various gas contents. When the quartz content is relatively high, there is a weak positive correlation with various gas contents. While feldspar and carbonate minerals have a negative correlation with various gas contents. This phenomenon provides a crucial basis for identifying high-quality reservoirs. In addition, as the thermal maturity of OM increases, variou gas contents show a significant upward trend. Therefore, a comprehensive consideration of reservoir characteristics such as geochemistry, mineralogy, and pore structure can not only promote the efficient development of continental shale gas resources but also provide theoretical guidance for future exploration work in similar basins.

## Data Availability

The datasets used to support the conclusions of this research can be obtained from the corresponding author upon reasonable request. However, they are not openly available due to confidentiality and ethical considerations.

## Abbreviations

TOC: Total organic carbon
XRD: X-ray diffraction
LTN_2_A: Low-temperature N_2_ adsorption
OM: Organic matter
PV: Pore volume
SSA: Specific surface area
APS: Average pore size
FESEM: Field emission scanning electron microscopy
PSD: Pore size distribution
IntraG: Intragranular
InterG: Intergranular
I/S: Illite/smectite
